# The effects of taxing sugar-sweetened beverages in Ecuador: An analysis across different income and consumption groups

**DOI:** 10.1371/journal.pone.0240546

**Published:** 2020-10-13

**Authors:** Joselin Segovia, Mercy Orellana, Juan Pablo Sarmiento, Darwin Carchi

**Affiliations:** 1 Grupo de Investigación en Economía Regional (GIER), Facultad de Ciencias Económicas y Administrativas, Universidad de Cuenca, Cuenca, Azuay, Ecuador; 2 Facultad de Ciencias Económicas y Administrativas, Universidad de Cuenca, Cuenca, Azuay, Ecuador; Xiamen University, CHINA

## Abstract

To analyze the effects of taxing sugar-sweetened beverages (SSBs) in Ecuador, this study estimates a Quadratic Almost Ideal Demand System model using data from the 2011–2012 National Survey of Income and Expenditure for Urban and Rural Households. We derive own- and cross-price elasticities by income quintiles and consumption deciles for five beverages, including two types of sugary drink: (i) milk, (ii) soft drinks, (iii) water, (iv) other sugary drinks, and (v) coffee and tea. Overall, results show that a 20% increase in the price of SSBs will decrease the consumption of soft drinks and other sugary drinks by 27% and 22%, respectively. Heterogeneous consumer behavior is revealed across income and consumption groups, as well as policy-relevant complementarity and substitution patterns. Policy impacts are simulated by considering an 18 cents per liter tax, implemented in Ecuador, and an ad-valorem 20% tax on the price. Estimated tax revenues and weight loss are larger for the latter. From a health perspective, high-income and heavy consumer households would benefit the most from this policy. Our study supports an evidence-based debate on how to correctly design and monitor food policy.

## Introduction

Overweight and obesity are considered a serious public health challenge, having a direct effect on quality of life as well as an indirect effect due to their relationship with non-communicable diseases (NCDs) [1–3]. In middle-income countries, rising overweight is coupled with consumption trends showing an increase in the intake of sugar-sweetened beverages (SSBs) [4, 5], which have been labelled a strong risk factor for type-II diabetes and cardiovascular disease [6–9]. Ecuador is a middle-income country with problematic nutrition habits, where nearly 30% of the population is estimated to excessively consume carbohydrates [10]. As a result, 6 in 10 adults suffer either obesity (22%) or overweight (40%); furthermore, NCDs represent the leading cause of death in the country, with type II diabetes playing a major role. The monthly average consumption of sugar-sweetened beverages in Ecuador amounts to approximately 5 liters per capita, and it can increase up to 60% in high-income households according to the 2012 National Survey of Income and Expenditure for Urban and Rural Households (ENIGHUR by its Spanish acronym) [11].

In light of this, Ecuador has adopted some of the health strategies recommended by the World Health Organization (WHO) [12], introducing traffic light front-of-pack labelling by the end of 2014 and a tax on sugary drinks in 2016 [13]. The aim of the latter is to discourage consumption and reduce overweight as a result, while also providing fiscal revenues to address the treatment and prevention of related diseases. A report by the WHO claims that despite facing political and industry opposition, taxes have proven to be effective at reducing consumption as long as they raise prices by 20% [14]. In Ecuador, a tax of 0.18 cents is levied on all sugary beverages with more than 25 grams of sugar per liter.

Several studies have been carried out using different approaches to evaluate the impact of taxes on consumption. Among these, there have been experimental, real-world, and simulation-based analyses [15]. The latter consists of estimating the consumer response to price changes generated by the tax [14], known as elasticities. An early meta-analysis found that the pooled price-elasticity coefficient is -1.299 [16], implying a 26% reduction in consumption given an increase of 20% in the price of SSBs. Systematic reviews have found own-price elasticities from -0.8 to -1.0 in high-income countries [15] and from -0.6 to -1.2 in middle-income countries [17], as well as larger estimates such as -2.255 [18] and 2.206 [19].

Like other countries implementing this fiscal measure, Ecuador lacks formal evaluations, mainly due to data unavailability. In fact, the latest comprehensive dataset on consumption and expenditure that is publicly available is the ENIGHUR 2012. In a previous study [20], price elasticities in Ecuador were found to fall between -1.17 and -1.33 for different income groups. However, one major drawback of this contribution is that it accounts for only two broad types of beverages: sugary and non-sugary drinks, limiting a detailed overview of potential substitution and complementarity patterns, which is key to determine the effectiveness of the tax [14].

To fill this gap, we estimate a demand system for five non-alcoholic beverages, including two types of sugar-sweetened beverages and three types of non-sugar drinks. We evaluate whether taxes are effective at discouraging SSB consumption by deriving own-price elasticities; and we analyze whether there are substitutive or complementary relationships that the tax may also trigger by deriving cross-price elasticities. With the aim of increasing the reliability of our results, we also take into account methodological issues usually found to generate biases, namely censoring and endogeneity [21–23]. Furthermore, we simulate the policy effects over tax collection and caloric intake variation. Results from our study help identify heterogeneity in consumption behavior across beverages as well as income and consumption groups and support the estimation of elasticities to analyze the effects of the tax policy.

## Materials and methods

### Data and variables

The estimation of the demand system and price elasticities is carried out using the National Survey of Income and Expenditure for Urban and Rural Households (ENIGHUR), collected between 2011 and 2012 by the National Institute of Statistics and Census (INEC) [11]. ENIGHUR is a cross-sectional survey that provides information on the amount, distribution, and structure of rural and urban household income and expenditure. It also provides a variety of social and demographic variables useful for the purpose of our study. ENIGHUR is obtained by applying a probabilistic, stratified, two-stage sample design to 39,617 households, and it is representative at the national, urban/rural, and regional and provincial levels. We limit our sample to those households consuming at least one product included in our analysis, resulting in a sample size of 32,191 households (81% of the original).

We aggregate purchases over 5 non-alcoholic beverage categories: (i) milk, which includes all types of low- and high-fat, plain, unflavored milk; (ii) soft drink SSBs, including regular soda drinks; (iii) water, including purified and sparkling/non-sparkling bottled water; (iv) other SSBs, a category that includes other sugar drinks such as fruit and vegetable drinks, tonic water, hydration and energy drinks; (v) coffee and tea, including roast, ground, and soluble coffee, tea bags, and herbal leaves. These categories are selected based on the literature and with the aim of disentangling demand behavior between sugar and non-sugar drinks. The former are subjected to a taxing policy and include the categories (ii) soft drink SSBs and (iv) other SSBs. We exclude from our analysis light soft drinks and concentrated juices since these entail two additional categories, but few households were found to consume them, implying a large percentage of zero consumption and making them unsuitable for analysis.

Quantities for all beverages are standardized in liters. Products within category five were originally registered in liters or grams. The latter are transformed first into cups by taking information provided by large chain supermarkets, cups are then converted into liters at the standard rate of 250 milliliters per cup. Expenditure data are expressed in U.S. dollars. Budget shares are calculated as the ratios between total expenditure by category and total expenditure on all 5 non-alcoholic beverage categories. Unit values of prices are generated by taking the ratio between expenditure and the quantity of each product consumed before aggregation into categories. Next, a price index is obtained based on the weighted arithmetic mean by category, which helps estimate price at the canton level for 209 cantons.

### Quadratic Almost Ideal Demand System (QUAIDS)

Our aim is to estimate demand elasticities to identify how price-responsive the consumption of SSBs is. In light of this, we first estimate a demand system extension of the Almost Ideal Demand System (AIDS) model [24] developed by [25], known as the Quadratic AIDS (QUAIDS). In sum, QUAIDS approaches consumption through household expenditure and introduces a quadratic term for income, relaxing the assumption of linearity of income-expenditure Engel curves and allowing for flexibility.

QUAIDS works under the assumption of weak budget separability, allowing for the estimation of total household demand divided into consumption categories—5 in our case. Furthermore, QUAIDS works within the framework of conditional demand systems. These face important limitations: (i) welfare measures may be biased, (ii) overall utility maximization can be violated, and (iii) the effect of changes in overall expenditure generated by price variations are omitted in the estimated elasticities [26]. In the context of this study, the latter implies that significant substitution elasticities with goods outside of the system might not be detected [27]. In spite of this, we choose QUAIDS given its properties and advantages over simpler models and their wide application in a variety of areas [e.g. 21, 25, 28–31].

QUAIDS departs from the indirect utility function of a PIGLOG demand system and models household expenditure patterns on a group of related items within a system of *i* goods. For a detailed derivation of the demand system, see [25]. The budget share, *w*_*i*_, for beverage *i* and household *h* is defined as follows:

$$w_{i}=\alpha_{i}^{\prime }+\sum_{j\in I}\gamma_{ij}\ln p_{j}+\beta_{i}\ln\left\{\frac{m}{a(P)}\right\}+\frac{\lambda_{i}}{b(P)}{\left[\ln\left\{\frac{m}{a(P)}\right\}\right]}^{2}\;\forall i\in I,$$ (1)

$$\alpha_{i}^{\prime }=\alpha_{i}+\sum_{k\in K}\rho_{ik}z_{k},$$ (2)

$$\ln a(P)=\alpha_{0}+\sum_{j\in I}\alpha_{j}\ln p_{j}+\frac{1}{2}\sum_{l\in I}\sum_{j\in I}\gamma_{lj}\ln p_{l}\ln p_{j},$$ (3)

$$b(P)=\prod_{i\in I}p_{i}^{\beta_{i}}.$$ (4)

For simplicity, we avoid subscript *h*. *p*_*i*_ represents the price of beverage *i*, while *m* is the total household expenditure on all beverages included in the system. *a*(*P*) is the cost of subsistence, approached by the translog price aggregator, and *b*(*P*) is the cost of bliss, approached by the Cobb–Douglas price aggregator. To model household heterogeneity, *z*_*k*_ includes a set of socio-demographic variables. *α*_*i*_,*β*_*i*_,*γ*_*lj*_,*ρ*_*ik*_ and *λ*_*i*_ are parameters to be estimated. The latter in particular, if statistically significant, allows us to determine whether the Engel curves are nonlinear. If not significant, Eq (1) turns into the linear version (AIDS). Eqs (1) to (4) entail that in the absence of variations in relative prices and real expenditure $\left(\frac{m}{a(P)}\right)$, the budget shares remain constant.

For this model to be consistent with consumer economic theory, some required properties of demand are fulfilled by imposing a set of linear restrictions over the parameters [32]. These include (i) homogeneity of degree zero on prices, which prescribes that given a proportional change in prices and income, demand remains unaffected, and (ii) symmetry of the Slutsky matrix, according to which a (complementarity or substitution) relationship between good *i* and *j* holds between *j* and *i*. The demand system is furthermore required to sum up to one given that it works with budget shares ∑_*i*∈*I*_ *w*_*i*_ = 1. The constraints over parameters are summarized as follows:

$$\sum_{i\in I}\alpha_{i}=1;\;\sum_{i\in I}\beta_{i}=0;\;\sum_{i\in I}\gamma_{ij}=0;\;\sum_{i\in I}\lambda_{i}=0;\;\forall i\in I,$$ (5)

$$\sum_{j\in I}\gamma_{ij}=0\;\forall j\in I;\;\gamma_{ij}=\gamma_{ji}\;\forall i,j\in I,$$ (6)

$$\sum_{i\in I}\rho_{ik}=0\;\forall k\in K.$$ (7)

### Endogeneity issue

Endogeneity affects prices and expenditure. First, expenditure might be endogenous given that budget shares (on the left-hand side of Eq (1)) and total expenditure (on the right-hand side) are mutually determined. On the other hand, the price might be endogenous because it is approached by unit values, since prices are not reported by many expenditure surveys such as ENIGHUR. Unit values, in turn, pose an endogeneity problem because they can be affected by measurement error and by quality effects due to heterogeneity in the preferences [32, 33].

Several methodologies have been proposed to correct for unit value endogeneity. These typically assume that geographically and time-clustered households face similar prices [32, 34–36]. We opt to use the two-step approach by [36], which simplifies the procedure for functional forms such as QUAIDS and is appropriate for our dataset since it is based on the median rather than on the mean unit value. First, the following equation is estimated:

$$v_{i}^{hsc}-{\left(v_{i}^{sc}\right)}_{med}=\mu_{i}D_{s}+\vartheta_{i}D_{c}+\phi_{i}x^{hsc}+\sum_{l\in L}\eta_{il}z_{l}^{hsc}+u_{i}^{hsc},$$ (8)

where $v_{i}^{hsc}$ represents the unit value paid by household *h* for beverage *i* in area *s* and canton *c*, while ${\left(v_{i}^{sc}\right)}_{med}$ is the median unit value in canton *c* and area *s*. Furthermore, *x*^*hsc*^ stands for per capita expenditure in food, and $z_{l}^{hsc}$ represents a set of household characteristics relevant to quality choice. In the latter, we include information of the household head such as age, education level and sex, and household information such as size and the percentage of women and adults older than 65. *D*_*s*_ and *D*_*c*_ are area and canton categorical variables, where *D*_*s*_ = 1 represents urban households. Lastly, $u_{i}^{hsc}$ is an error term, and *μ*_*i*_, *ϑ*_*i*_, *ϕ*_*i*_ and *η*_*il*_ are unknown parameters. Unlike [36], we do not include the share of beverages consumed away from home due to the absence of this information in our data source. Hence, our study is representative of household consumption only.

The second stage consists of obtaining the quality-adjusted unit values by adding the canton median unit value to the residual estimated in Eq (8) as follows:

$${\left(p_{i}^{sc}\right)}_{med}={\left(v_{i}^{sc}\right)}_{med}+{\left({\hat{u}}_{i}^{sc}\right)}_{med}.$$ (9)

Through this procedure, unit values are adjusted by quality and demographics by first estimating Eqs (8) and using (9). These are then introduced as prices in the demand estimation.

Furthermore, to correct for endogeneity in expenditure data, we adopt the control-function approach of [28] and [29], where we regress the log of total expenditure on a set of explanatory variables. Residuals are then estimated and introduced as an explanatory variable in the demand system. As part of our explanatory variables, we include the geographic area (rural/urban), age, sex, and education level of the householder, and household size. We also calculate an index of access to basic services as a key variable in Ecuador (piped water, electricity, and sanitation). This approach has been proven to be the most appropriate for nonlinear regression models, leading to consistent estimates [37].

### Censoring issue

Censoring arises when households report zero expenditure due to multiple reasons related to the consumer (e.g. not preferring the good or non-affordability) and other reasons related to the non-availability of the product or the inability of the survey’s timeframe to capture that type of consumption. Ignoring censoring can lead to selection bias; therefore, we account for this by adopting the two-step approach of [38], as widely used in the literature [21, 28, 29, 39]. This approach is selected over other similar methods, such as that of [40] based on the Heckman estimator, because it is the most appropriate when the full sample is available and includes limit and no-limit observations, that is, censored data such as ours.

The method in [38] assumes that household consumption is a two-step decision. In the first stage, households decide whether or not to consume the good, which can be represented by the following equation:

$$D_{i}^{*}=\sum_{j\in I}\tau_{j}\;\ln\;p_{j}+\pi_{i}m+\sum_{k\in K}\theta_{k}z_{k}+\varepsilon_{i},$$ (10)

$$D_{i}=\left\{ \begin{array}{c} 0\;if\;D_{i}^{*}\leq 0\\ 1\;if\;D_{i}^{*}>0 \end{array}.\right.$$ (11)

Subscript *i* represents the beverage category. $D_{i}^{*}$ is a latent non-observable variable, while ε_i_ is a residual term. Variable *D*_*i*_ is observed according to the rule expressed in (11) and takes a value of one if the household reported positive consumption of beverage *i*, and zero if not.

In the second stage, households choose the budget share for each beverage category, conditioned upon positive consumption in the first stage. It is considered that the distribution of the censored variable, $w_{i}^{0}$, is a mixture of a continuous distribution (in case $D_{i}^{*}>0$) and a discrete distribution (in case $D_{i}^{*}\leq 0)$, leading to

$$w_{i}^{0}=D_{i}w_{i}^{*},$$ (12)

where

$$w_{i}^{*}=\Phi_{i}w_{i}+\delta_{i}\phi_{i}+\xi_{i}.$$ (13)

Variable $w_{i}^{*}$ describes the latent counterpart of $w_{i}^{0}$, and *ξ*_*i*_ is an error term. Estimation consists of two steps. First, a discrete choice model for the decision of consumption is estimated by means of a probit function. From this estimation, the cumulative distribution (*Φ*) and probability density function (*ϕ*) are predicted. In the second step, these variables are introduced into the demand system that will be estimated:

$$w_{i}^{*}={\hat{\Phi }}_{i}w_{i}+\delta_{i}{\hat{\phi }}_{i}+\xi_{i}\;\forall i\in I$$ (14)

Estimation of the system (14) through nonlinear seemingly unrelated regression (NLSUR) generates consistent estimates. However, these are inefficient given that the error term is heteroskedastic and, as noted by [38], the covariance matrix of the second-step estimator is incorrect. We correct this by bootstrapping the standard errors.

### Price elasticities

The censoring-adjusted price elasticities are estimated through the following expression [30] at the sample means of the variables:

$$\epsilon_{ij}=-\delta_{ij}+\frac{1}{w_{i}^{*}}[\Phi_{i}(\gamma_{ij}-(\beta_{i}+\frac{2\lambda_{i}}{b(P)}\ln\left(\frac{m}{a(P)}\right))*\left(\alpha_{j}+\sum_{k}\gamma_{jk}\ln p_{k}\right)-\frac{(\beta_{j})\lambda_{i}}{b(P)}({\ln\left(\frac{m}{{\bar{m}}_{0}(z)a(P)}\right)}^{2}))+\tau_{j}\phi_{i}(w_{i}-\delta_{i}D_{i}^{*})],$$ (15)

where *δ*_*ij*_ is the Kronecker Delta, which takes a value of 1 for own-price elasticities and zero otherwise, while $D_{i}^{*}$ is the argument of the function defined in Eq (10). The elasticities estimates tell us whether consumption changes with price and the magnitude of the change. A coefficient higher (lower) than 1 in absolute value reveals that the variation in consumption will be higher (lower) than the variation in price, meaning that the demand is elastic (inelastic). The coefficient could also be equal to 1, in which case the demand is unitary elastic, implying proportional changes in quantity and price.

### Estimation strategy

To carry out the analysis, the decision to consume defined in Eqs (10) and (11) is estimated by means of a probit model. Following [25], we set *α*_0*i*_ = 5 and estimate Eq (14) using nonlinear seemingly unrelated regression (NSUR) through feasible generalized nonlinear least squares (FGNLS). To correct the covariance matrix in the system (14), the standard errors for the estimated parameters are bootstrapped with 200 replications. Furthermore, results are obtained by income quintiles and by SSB soft drinks consumption deciles. All estimations were carried out using Stata 15.

### Simulation of policy results

We simulate the policy results within two domains: (i) the collection of tax revenues for the first year that the tax was effective (2017) and (ii) the variation in consumed calories and, hence, potential weight effects. Tax collection is obtained for both types of sugary drink as the tax times the projected consumption. We apply a 20% tax on the price, following the WHO recommendations [41], and a volumetric tax of 18 cents per liter of sugary drink, corresponding to what was implemented in Ecuador. A full pass-through rate is considered as in [42–44], meaning that the retail price will be increased by the amount of the tax. The market for soft drinks in Ecuador is highly concentrated; therefore, we would expect this to be the case [20]. Average prices per beverage category are used, adjusted by inflation. Projected consumption is obtained combining consumption and elasticities. Results under both the volumetric and the ad valorem tax are compared with the real collection results reported by official data [45], with the aim of assessing the reliability of the methodology for analyzing policy results.

Furthermore, to simulate weight effects, the variation in calorie intake is obtained by linking nutritional information from the USDA’s nutrient database [46] to each beverage category before and after the tax. Assuming that 1 pound of body fat translates into approximately 3,500 calories, we estimate the change in per capita body weight as in [44, 46]. We present these results by income and consumption group.

## Results

In this section, we first show descriptive statistics of the variables included in our demand system. For simplicity, these are shown for 3 quintiles: low-, middle- and high-income households. Next, we briefly discuss the outcomes of the demand system estimation, and then we focus on presenting the results of the elasticities. Lastly, we present the estimated effects of the taxing policy.

Table 1 shows sociodemographic descriptive statistics. It is observed that lower-income households are more likely to live in rural areas. They are also larger and have younger and less-educated heads of household.

**Table 1:** Sociodemographic descriptive statistics: Whole sample and income quintiles.

*Variable*	*Quintile 1*	*Quintile 3*	*Quintile 5*	*Total sample*
*Number of households*	6,438	6,438	6,438	32,191
*Average per capita income ($/month)*	80.34	199.85	744.67	281.66
*Area (1 = urban)*	0.47	0.74	0.91	0.71
*Gender of household head (1 = male)*	0.80	0.76	0.75	0.77
*Age of head of household*	45.6	47.4	49.6	47.3
*Education of head of household*	6.5	8.8	13.7	9.3
*Household size*	5.3	3.9	2.8	4.0

Source: National Survey of Income and Expenditure for Urban and Rural Households 2011–2012. Ecuador. Weighted values.

Table 2 shows descriptive statistics regarding household consumption of and expenditure on non-alcoholic beverages. Expenditure shares shed some light on the relative importance of each beverage within the consumers’ budget and show different patterns across income groups. For instance, high-income households spend significantly more on milk and water than low-income households. In addition, most of their budget is spent on milk, but low-income families spend nearly equal shares on soft drinks and on milk.

**Table 2:** Expenditure and consumption descriptive statistics: Whole sample and income quintiles.

Beverage category	Quintile 1	Quintile 3	Quintile 5	Total sample
**Mean expenditure shares (%)**				
*Milk*	28.0%	34.6%	34.3%	32.9%
*SSBs—soft drinks*	28.8%	24.6%	19.5%	24.2%
*Water*	18.0%	18.8%	22.7%	19.7%
*SSBs—other*	9.4%	13.1%	18.7%	13.4%
*Coffee and tea*	15.8%	8.9%	4.8%	9.7%
**Mean unit values, quality adjusted ($/Liter)**				
*Milk*	0.70	0.74	0.76	0.74
*SSBs—soft drinks*	0.86	0.83	0.83	0.83
*Water*	0.65	0.65	0.66	0.65
*SSBs—other*	1.43	1.48	1.52	1.48
*Coffee and tea*	0.66	0.69	0.67	0.67
**Mean unit values per liter ($/Liter)**				
*Milk*	0.77	0.80	0.91	0.82
*SSBs—soft drinks*	0.97	0.95	1.01	0.97
*Water*	0.67	0.67	0.70	0.68
*SSBs—other*	1.42	1.59	1.76	1.61
*Coffee and tea*	0.70	0.70	0.58	0.68
**Mean per capita quantity (Liter/month)**				
*Milk*	1.40	3.08	5.07	3.14
*SSBs—soft drinks*	1.11	2.05	2.89	2.03
*Water*	0.83	1.92	4.60	2.37
*SSBs—other*	0.22	0.67	1.77	0.83
*Coffee and tea*	0.86	1.05	1.64	1.16

Source: National Survey of Income and Expenditure for Urban and Rural Households 2011–2012. Ecuador. Weighted values.

Considering unit values, soft drinks are the second most expensive type of non-alcoholic beverage after other sugary drinks, while water and coffee and tea are the least expensive. It is also interesting to note that except for coffee and tea, unit values paid by the 5th quintile are higher, showing a difference in buying patterns where perhaps quality or quantity play a role [32]. In fact, quality-adjusted prices support this claim. Furthermore, milk shows the highest per capita consumption overall and by income quintile, and soft drinks are the third most consumed beverage, despite considerable differences in consumption between low- and high-income families.

The outcomes of the demand system estimation can be found in S1 Table. All of the equations show high coefficients of determination, pointing out the high explanatory power of our covariates. The coefficient associated with parameter *λ* is statistically significant, meaning that the estimation of QUAIDS is preferred over the linear version, AIDS, in 4 out of 5 equations. In S1 Table, we also observe the coefficients associated with the demographic variables. These allow us to conclude, for instance, that soft drink expenditure decreases with age, education, and rural residence but increases with household size.

Table 3 reports the uncompensated price elasticities using the whole sample and accounting for censoring and potential endogeneity both in expenditures and in prices. Own-price elasticities are shown along the diagonal (in bold), and cross-price elasticities are shown off the diagonal. Most of our estimates are statistically different from zero. Out of 25 coefficients, 20 conform to this.

**Table 3:** Uncompensated price elasticities: Whole sample.

	Change in price				
Change in quantity	Milk	SSBs—soft drinks	Water	SSBs—other	Coffee and tea
**Milk**	-1.1805 ***	-0.1165	0.0403	0.3072 **	-0.0786 ***
	(0.0305)	(0.0252)	(0.0220)	(0.0237)	(0.0108)
**SSBs—soft drinks**	-0.1102 *	-1.3499 ***	0.2910 *	0.0337 ***	0.1447 ***
	(0.0361)	(0.0518)	(0.0340)	(0.0393)	(0.0135)
**Water**	0.1095	0.3902	-0.7434 ***	-0.5214 ***	-0.1722 ***
	(0.0335)	(0.0374)	(0.0406)	(0.0325)	(0.0145)
**SSBs—other**	0.5103 ***	-0.0768 ***	-0.6362 ***	-1.1143 ***	0.0581
	(0.0403)	(0.0495)	(0.0347)	(0.0570)	(0.0186)
**Coffee and tea**	-0.0253 *	0.5096 ***	-0.5326 ***	0.1093 *	-0.7613 ***
	(0.0452)	(0.0512)	(0.0399)	(0.0532)	(0.0327)

Source: National Survey of Income and Expenditure for Urban and Rural Households 2011–2012. Ecuador. Bold denotes own-price elasticities. Std. Err. in parentheses.

* p<0.05

** p<0.01

*** p<0.001.

Results from own-price elasticities point to an elastic demand behavior for three out of five consumption categories: milk, soft drinks, and other SSBs, while water and coffee and tea are inelastic. In particular, soft drinks present the highest elasticity coefficient in absolute terms (-1.35), which is an important finding for public policy, suggesting that an increase of 20% in the price of soft drinks is likely to decrease consumption by nearly 27%. This result is larger than the range suggested by [17], where it is found that for middle-income countries (MICs) elasticity usually falls between -0.6 and -1.2. However, most of the studies in this survey do not deal with censoring in data, which can introduce bias. In fact, depending on the model choice and the group aggregation, results in previous studies vary markedly [15, 16, 44, 47]. Methodologically, our finding is closer to [28], where SSB own-price elasticity estimates stand at 1.62 when accounting for censoring and endogeneity problems. In relation to the previous estimation for Ecuador [20], our elasticity coefficient is larger in absolute terms (-1.35 versus -1.20), but as mentioned earlier, these are not directly comparable since our research design differs in multiple significant aspects.

Given a price change in soft drink SSBs, other SSBs show negative cross-price elasticities, implying that they are complements in consumption. This relationship remains when looking at a price change for other SSBs. On the other hand, coffee and tea are found to be a substitute for SSBs. The complementarity of both sugar-based drinks is crucial to identify whether a tax policy would work or not. If they were substitutes, a price increase in one of them would only shift consumption towards the other type of SSB, undermining the efficiency of the policy. Previous literature with regards to this has been inconclusive, finding mixed results. In this sense, our results are in line with [44, 48, 49] but differ from [50].

Table 4 shows a comparison of own-price elasticity estimates without correcting for censoring and endogeneity (Model 1), correcting for endogeneity only (Model 2), and correcting for both methodological issues (Model 3). A complete overview of Model 1 is reported in S2 Table. In our case, ignoring these methodological issues leads to mixed results, as previously found [21, 23, 28]. In particular, estimates for milk and SSBs are downward-biased and estimates for water and coffee and tea are upward-biased. Estimates of the coefficients associated with censoring are highly significant, showing that censoring plays a role in our model (available upon request); therefore, we adopt corrected estimates (Table 3) for policy simulations and discussion throughout the rest of the document.

**Table 4:** Uncompensated own-price elasticities, corrected and uncorrected models: Whole sample.

Beverage category	Model 1	Model 2	Model 3
**Milk**	-0.9863 ***	-1.1341 ***	-1.1805 ***
	(0.0217)	(0.0196)	(0.0305)
**SSBs—soft drinks**	-1.2931 ***	-1.3777 ***	-1.3499 ***
	(0.0237)	(0.0391)	(0.0518)
**Water**	-0.9277 ***	-0.9038 ***	-0.7434 ***
	(0.0198)	(0.0281)	(0.0406)
**SSBs—other**	-0.7087 ***	-1.1585 ***	-1.1143 ***
	(0.0336)	(0.0438)	(0.0570)
**Coffee and tea**	-1.0200 ***	-0.8334 ***	-0.7613 ***
	(0.0246)	(0.0212)	(0.0327)

Source: National Survey of Income and Expenditure for Urban and Rural Households 2011–2012. Ecuador. Std. Err. in parentheses.

* p<0.05

** p<0.01

*** p<0.001.

**Fig 1:**
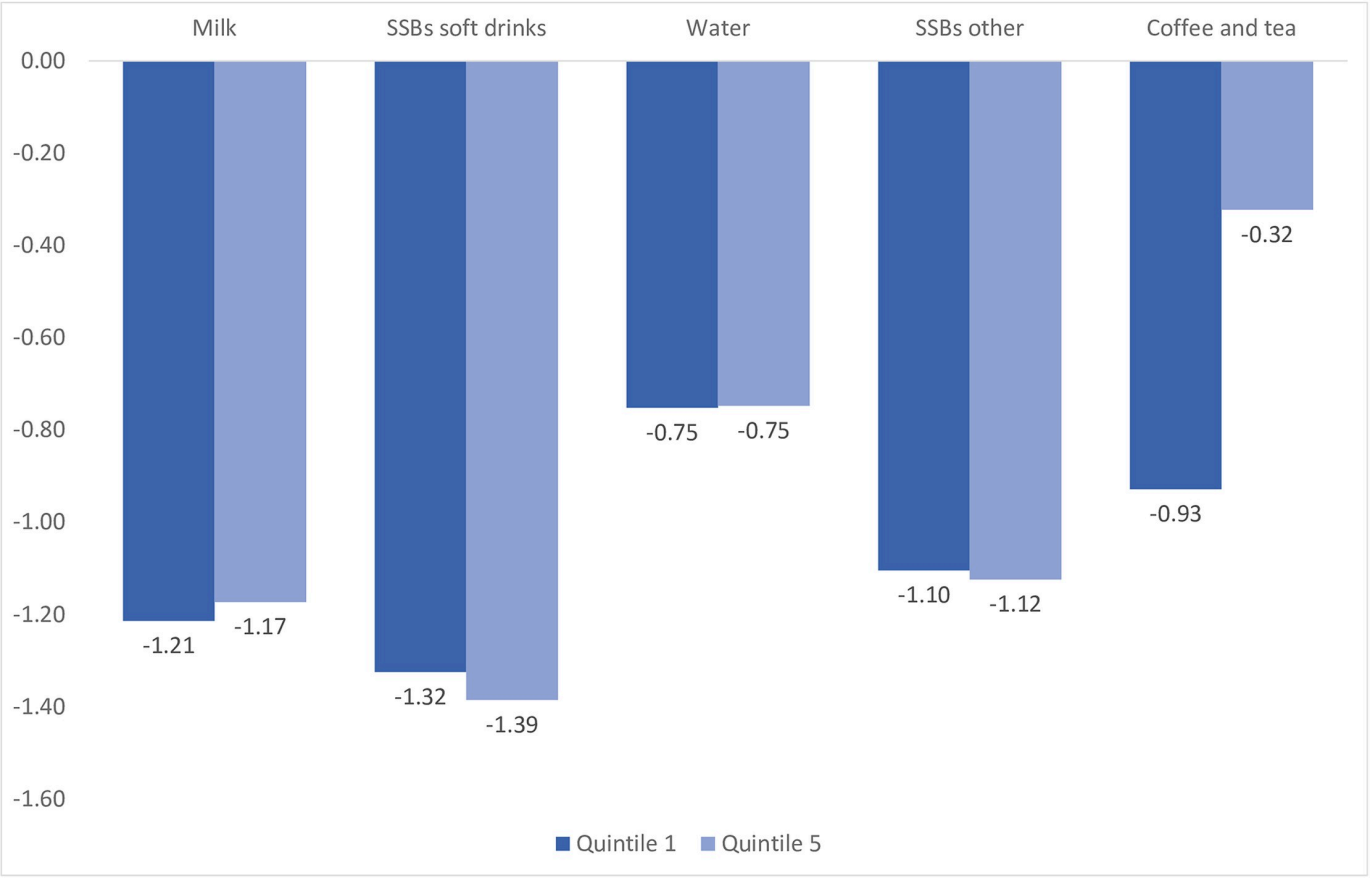
Uncompensated own-price elasticities: High- and low-income consumers. Source: National Survey of Income and Expenditure for Urban and Rural Households 2011–2012. Ecuador.

The estimation of own- and cross-price elasticities by income quintiles presents similar patterns to what has already been discussed in terms of which beverages are elastic and inelastic. For ease of presentation, we report only own-price elasticities for low-income and high-income households in Fig 1, while cross-price elasticities are reported in S3 and S4 Tables. The estimates for all quintiles are available upon request. Overall, results show differences in the response to price changes between low- and high-income households, except for water consumption. For the rest of the beverages, demand behavior shows different patterns. For instance, it is more elastic for high-income households for both SSBs but less elastic for milk and coffee and tea. These patterns are in line with [28, 29, 44, 48], except for soft drinks, which are usually found to be less elastic in high-income households. The latter could be explained by the fact that expenditure on soft drinks is relatively less important for this group. As reported in Table 2, their budget share for soft drinks is approximately 10 percentage points lower than for low-income households.

Results for complementarity and substitution patterns are heterogeneous. For instance, given a price increase in soft drinks, the effect on other SSBs intake is lower for the lowest income group, but the effect on milk intake is higher.

**Fig 2:**
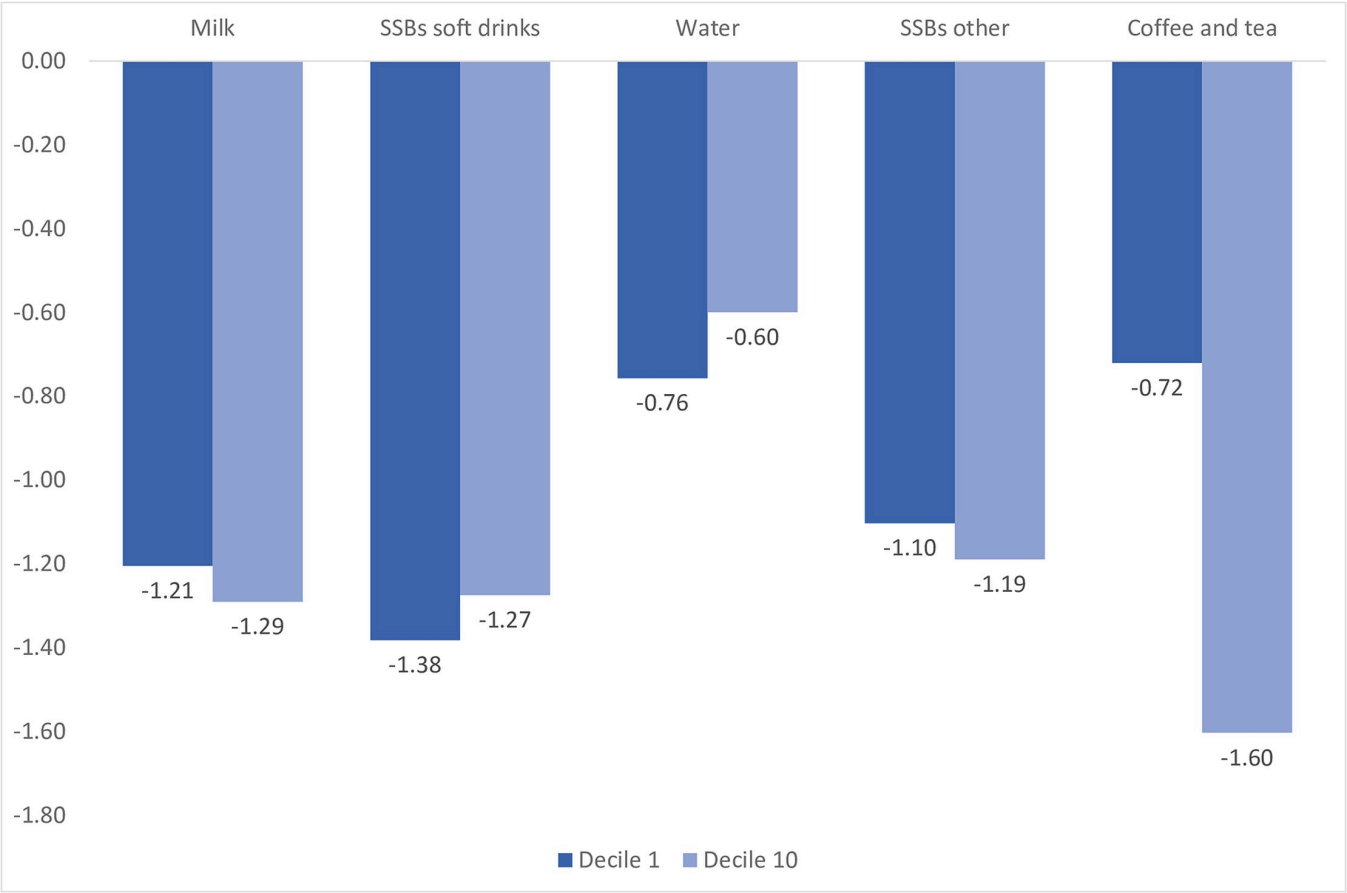
Uncompensated own-price elasticities: Light and heavy consumers. Source: National Survey of Income and Expenditure for Urban and Rural Households 2011–2012. Ecuador.

Own-price elasticities derived at consumption deciles for soft drinks are shown only for the 1st (light consumers) and 10th decile (heavy consumers) in Fig 2, while cross-price elasticities can be found in S5 and S6 Tables. Results for the deciles 2 to 9 are available upon request. Similar to the estimates for the whole sample, water and coffee and tea remain inelastic for light consumers, but coffee and tea become elastic for heavy consumers. These results show some variation in magnitude between consumption groups. Notably, light consumers are more responsive to the price changes of soft drinks and water than heavy consumers.

Lastly, the simulated effects of an ad-valorem tax (20% on the price) and the existing volumetric tax (18 cents per liter) on SSBs are shown in Table 5. Official tax revenues for the first year that the volumetric tax was effective in Ecuador are also shown. Contrary to the recommendation by [14], the existing tax represents nearly 15% of the price for soft drinks and only 9% for other SSBs. Accordingly, its effects on consumption and tax collection are lower.

**Table 5:** Policy impact simulation: Revenues and caloric change, whole sample.

	Ad-valorem tax 20% on price	Volumetric tax 18 cents per liter	Volumetric tax 18 cents (official data)
Revenues	$150,081,716	$104,397,400	$118,261,682
Calories: SSBs—soft drinks	-3,779	-2,794	
Calories: SSBs—others	-2,360	-1,133	
Indirect effect on calories	1,599	748	
Total effect on calories	-4,540	-3,180	

Source: Own estimates based on Table 3. Tax collection information is provided by the Servicio de Rentas Internas del Ecuador, 2020. Indirect effect represents the effects of cross-price elasticities.

The ad-valorem tax would, in fact, discourage soft drink demand by 26% and other SSBs by nearly 24%. This would translate to a reduction of approximately 4,500 annual calories, leading to a reduction in body weight of 0.58 kg for an average adult, which falls in the range of [44, 46, 51]. Furthermore, the tax would yield revenues that average 0.64% of the total fiscal revenue for the Ecuadorian government in 2017 [52]. This falls in the range of previous results for Latin American countries such as Chile, 0.47% [29], and Colombia, 1% [28]. On the other hand, the volumetric tax would reduce annual caloric intake by 3,100 calories and would yield revenues accounting for 0.44% of the total fiscal revenue. In both scenarios, results would be mainly driven by the impact on soft drinks because per capita consumption is higher but it is also more widespread across the population. While 65% of households report soft drink consumption, 45% consume other types of SSBs.

When comparing our estimation to the official revenue, we observe a gap that could be attributed to consumption away from home, which was excluded from our study due to data unavailability. This consumption in Ecuador represents 20% of the total consumption of non-alcoholic beverages [11].

**Fig 3:**
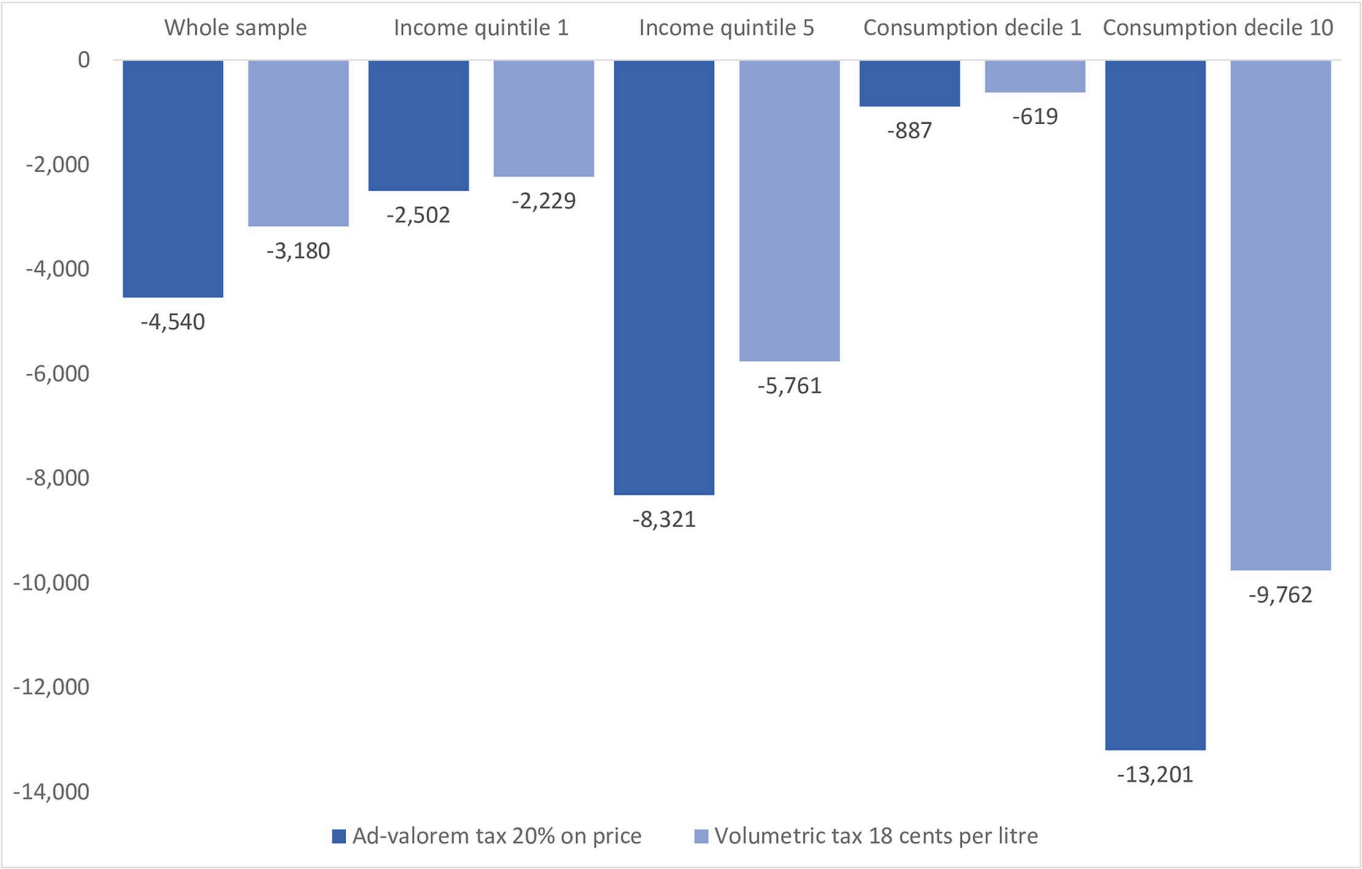
Policy impact simulation: Caloric change across income and consumption groups. Source: Own estimates based on Table 3 and S3–S6 Tables.

Fig 3 gives insight into the diverse impacts that the policy could have across income and consumption groups. Still, the ad-valorem tax produces greater effects, but these clearly differ from the simulation for the whole sample. Higher income households and heavy consumers would benefit the most in terms of weight reduction, with a reduction of 8,320 and 13,200 annual calories. This translates into a reduction of 1.08 and 1.71 kg in body weight, closer to the results for heavy consumption countries such as the U.S. [46].

Although the focus of this study is to analyze the implications of taxing SSBs, to conclude this section we briefly review other valuable results that the analysis allows us to identify. As an example, milk is found to be elastic, which implies that its consumption varies more than proportionately to its price changes. Furthermore, if milk prices change, soft drinks appear to be a complement whereas other SSBs appear to be a substitute. The latter effect is relatively larger, indicating that milk intake might be threatened if it incurs a price increase in Ecuador. Results show that a 10% increase in milk price will decrease its consumption by 12% and will shift this mostly towards other SSBs. Taking a closer look by income level (S3 and S4 Tables), this substitution effect is larger for lower-income consumers.

## Discussion

Given the lack of studies investigating the impact of the tax levied on sugar-sweetened drinks in Ecuador, the aim of this study was to offer insight into this. Potential effects were approached via elasticity estimates derived from a Quadratic Almost Ideal Demand System for five non-alcoholic beverages, corrected for censoring and endogeneity.

We carried out our analysis of elasticities for an overall sample of Ecuadorian households, as well as by income quintiles and consumption deciles. The elasticity coefficients show variation by income and consumption group, revealing differences in consumer behavior across the population. All of our estimates indicate that milk, soft drinks, and other SSBs are elastic, while water and coffee and tea are inelastic.

The demand for both soft drinks and other sugar-sweetened drinks is found to be price-elastic, in line with the literature [16, 17, 28, 29, 44, 48]. With respect to previous evidence for Ecuador [20], our results show that a lower aggregation of products allows us to identify relevant heterogeneous elasticity patterns between beverages.

In terms of public policy, our paper unveils five major results. First, we observed that a taxing policy would be effective at reducing the consumption of both types of SSBs due to their elastic demand behavior. Another reason for this is based on cross-price elasticities, which show that the effect of a tax on either type of sugary drink will be reinforced by its symmetric complementarity [44, 48, 49].

A second finding that draws attention is associated with the robust complementarity of both types of sugar-sweetened beverages included in our study. This entails that they are positively related and consumed together, irrespective of income or consumption levels. Such a finding suggests that, in the more generalized scenario, one unhealthy habit might be likely to lead to another one. This finding, therefore, leaves some open questions for future research with regards to relationships with other harmful consumption habits beyond SSBs. In the case of beverages, our claim is supported by our dataset, showing that low (high) soft drink intake results in a low (high) intake of other sugar drinks. This result, in turn, implies some optimism for the potential to improve consumer health from a public policy perspective.

Another important result is related to the analysis of the relationship between SSBs and other beverages through cross-price elasticities. We observe that while a tax on SSBs soft drinks has no statistically significant impact on necessary beverages such as water and milk, a tax on other SSBs is likely to decrease water intake by approximately 10%. It is crucial to analyze these types of effects since the proper design of a policy largely relies on identifying its undesirable or adverse indirect effects [14]. The underlying mechanism behind this outcome is beyond the scope of this study but certainly deserves more investigation, especially for deprived and low-income households.

A fourth relevant finding refers to the behavior of the demand for milk. It is elastic in all of our estimations, in line with [28, 29, 44, 48]. Furthermore, we observe that its consumption would shift towards other SSBs and be displaced by price changes in soft drinks. Both effects are larger for low-income consumers. Taken together, these results give us insight into the claim that in low-to-middle-income countries, milk consumption might be seriously threatened by price changes and be displaced by SSB consumption, potentially reducing the intake of key nutrients and vitamins. Therefore, these findings are important for policy design and monitoring and take particular relevance in countries where children’s stunting and overweight coexist [17], as is the case in Ecuador [10].

One last finding is derived from the simulation of tax collection. It differs from the official revenues by a magnitude that could be attributed to consumption away from home, which was excluded from our work. This demonstrates that the demand system estimation is a powerful tool for public policy design and analysis. Regarding the estimation of the caloric and weight impact, to our knowledge no study has been carried out in Ecuador; therefore, our study contributes to filling this gap. As discussed, the effects on body weight are small for the overall sample, but it should be noted that even for this range of subtle changes, significant positive changes in obesity and diabetes rates can be expected [51, 53]. These effects would be stronger for high-income and, in particular, heavy consumption households. Despite their consumer response subtly differing from the average consumer, any change becomes more significant given their high intake of sugary drinks. It is important to remark that the estimated consumption impact is likely to be sustained through several years [54].

Based on this and the simulation of the two types of tax, we argue that the country would benefit from adopting a policy that follows the recommendations of the World Health Organization. This tax would generate greater impacts on body weight and would allow for higher revenues to treat obesity and overweight. However, we emphasize that the effects on body weight may be overestimated given that our demand system follows a conditional approach [27]. Unfortunately, no previous study on this matter has been conducted; hence, we have no counterfactual data as in the case of tax revenues.

This study presents some limitations. First, the results are representative for at-home consumption since data for away-from-home consumption is not available. It is highly likely that behavior away from home might be driven by different factors, giving rise to variation in the response to price changes, and this might take particular relevance in contexts where consumption is mostly carried out away from home. In our case, however, at-home consumption is the most common, representing nearly 80% of total consumption [11]. Second, our model does not include possible substitution or complementarity patterns beyond beverages. It is possible that sugar snacks or high-fat foods are a substitute for SSBs, in which case a tax will shift consumption towards those products, jeopardizing the effects of the policy. On the other hand, they may be complementary goods, increasing the power of the tax to decrease the consumption of other unhealthy products. Evidence with regards to this has been inconclusive, showing mixed results [27–29, 55]. It is important to note that even if we include food in our demand system, the estimated caloric change could be biased by using a conditional demand system [27]. Moreover, the final consumption variation will be a result of multiple variables beyond this analysis, including consumer-side and industry-related factors.

## Conclusions

Ecuador has been a pioneer in the field of food regulation in Latin America [14], and it is one of the few countries in the region that has taxed SSBs to discourage their consumption. We found that taxing all types of SSBs might be effective. Our methodological approach has proven to be helpful for disentangling relationships across beverages, shedding light on critical issues in MICs, such as that of adequate milk intake. Furthermore, it has proven suitable for simulating results close to reality in terms of tax collection. Lastly, our results show that there is still room for improvement in this public policy domain by adjusting the tax to international recommendations. In consequence, our study supports an evidence-based debate on how to correctly design and monitor food policy.

## Supporting information

S1 Table Demand system estimation outcome. (DOCX)

S2 Table Uncompensated price elasticities: Whole sample. Uncorrected model.
(DOCX)

S3 Table Uncompensated own-price elasticities: First income quintile. (DOCX)

S4 Table Uncompensated own-price elasticities: Fifth income quintile. (DOCX)

S5 Table Uncompensated own-price elasticities: Light consumers. (DOCX)

S6 Table Uncompensated own-price elasticities: Heavy consumers. (DOCX)
