# Supplementary material for: The effects of taxing sugar-sweetened beverages in Ecuador: An analysis across different income and consumption groups
Source: PLoS One. 2020 Oct 13;15(10):e0240546. doi: 10.1371/journal.pone.0240546 (PMC7553359; doi:10.1371/journal.pone.0240546)
Supplement: S1 Table — (DOCX) [file pone.0240546.s001.docx]

**S1 Table. Demand system estimation outcome**

|  | **Equation** | **Obs** | **RMSE** | **R-sq** | **Constant** |  |
| --- | --- | --- | --- | --- | --- | --- |
| 1 | w1 | 32,191 | 0.2996222 | 0,5719* | (none) |  |
| 2 | w2 | 32,191 | 0.2655285 | 0,4442* | (none) |  |
| 3 | w3 | 32,191 | 0.2508024 | 0,4257* | (none) |  |
| 4 | w4 | 32,191 | 0.2081665 | 0,3104* | (none) |  |
| * Uncentered R-sq | |  |  |  |  |  |

|  | Observed Coef. | Bootstrap  Std. Err. | z | P>z | Normal based  [95% Conf. Interval] | |
| --- | --- | --- | --- | --- | --- | --- |
| **Alpha** |  |  |  |  |  |  |
| /a1 | -0.1309 | 0.0411 | -3.19 | 0.0010 | -0.2115 | -0.0504 |
| /a2 | 0.4873 | 0.0424 | 11.49 | 0.0000 | 0.4042 | 0.5705 |
| /a3 | 0.1911 | 0.0417 | 4.58 | 0.0000 | 0.1093 | 0.2729 |
| /a4 | 0.6749 | 0.0480 | 14.07 | 0.0000 | 0.5808 | 0.7689 |
| Beta |  |  |  |  |  |  |
| /b1 | -0.1767 | 0.0122 | -14.51 | 0.0000 | -0.2006 | -0.1529 |
| /b2 | 0.0057 | 0.0121 | 0.47 | 0.6350 | -0.0180 | 0.0295 |
| /b3 | 0.0439 | 0.0136 | 3.23 | 0.0010 | 0.0172 | 0.0705 |
| /b4 | 0.2278 | 0.0139 | 16.39 | 0.0000 | 0.2006 | 0.2551 |
| **Gamma** |  |  |  |  |  |  |
| /g11 | -0.0516 | 0.0160 | -3.22 | 0.0010 | -0.0829 | -0.0202 |
| /g12 | -0.0405 | 0.0126 | -3.22 | 0.0010 | -0.0652 | -0.0159 |
| /g13 | 0.0176 | 0.0110 | 1.60 | 0.1090 | -0.0039 | 0.0390 |
| /g14 | 0.1053 | 0.0138 | 7.65 | 0.0000 | 0.0783 | 0.1323 |
| /g22 | -0.1242 | 0.0179 | -6.92 | 0.0000 | -0.1593 | -0.0890 |
| /g23 | 0.1024 | 0.0119 | 8.63 | 0.0000 | 0.0791 | 0.1256 |
| /g24 | 0.0120 | 0.0141 | 0.85 | 0.3960 | -0.0157 | 0.0396 |
| /g33 | 0.0806 | 0.0129 | 6.24 | 0.0000 | 0.0553 | 0.1059 |
| /g34 | -0.1647 | 0.0108 | -15.22 | 0.0000 | -0.1859 | -0.1435 |
| /g44 | 0.0656 | 0.0207 | 3.17 | 0.0020 | 0.0250 | 0.1063 |
| **Lambda** |  |  |  |  |  |  |
| /l1 | -0.0459 | 0.0027 | -17.00 | 0.0000 | -0.0512 | -0.0406 |
| /l2 | 0.0022 | 0.0029 | 0.77 | 0.4410 | -0.0034 | 0.0078 |
| /l3 | 0.0200 | 0.0032 | 6.33 | 0.0000 | 0.0138 | 0.0262 |
| /l4 | 0.0384 | 0.0035 | 10.95 | 0.0000 | 0.0316 | 0.0453 |
| **Rho** |  |  |  |  |  |  |
| /r11 | 0.0972 | 0.0103 | 9.41 | 0.0000 | 0.0769 | 0.1174 |
| /r21 | -0.0759 | 0.0058 | -12.98 | 0.0000 | -0.0874 | -0.0644 |
| /r31 | 0.0475 | 0.0083 | 5.73 | 0.0000 | 0.0312 | 0.0638 |
| /r41 | 0.0071 | 0.0013 | 5.23 | 0.0000 | 0.0044 | 0.0097 |
| /r51 | -0.0181 | 0.0055 | -3.28 | 0.0010 | -0.0289 | -0.0073 |
| /r61 | -0.0158 | 0.0071 | -2.23 | 0.0260 | -0.0297 | -0.0019 |
| /r12 | -0.1265 | 0.0067 | -19.00 | 0.0000 | -0.1396 | -0.1135 |
| /r22 | 0.0346 | 0.0064 | 5.42 | 0.0000 | 0.0221 | 0.0472 |
| /r32 | -0.0565 | 0.0087 | -6.48 | 0.0000 | -0.0735 | -0.0394 |
| /r42 | -0.0117 | 0.0010 | -11.56 | 0.0000 | -0.0137 | -0.0097 |
| /r52 | 0.0125 | 0.0065 | 1.92 | 0.0540 | -0.0002 | 0.0252 |
| /r62 | -0.0196 | 0.0069 | -2.83 | 0.0050 | -0.0331 | -0.0060 |
| /r13 | 0.0011 | 0.0066 | 0.17 | 0.8690 | -0.0118 | 0.0140 |
| /r23 | 0.0344 | 0.0054 | 6.38 | 0.0000 | 0.0238 | 0.0449 |
| /r33 | 0.0382 | 0.0083 | 4.62 | 0.0000 | 0.0220 | 0.0544 |
| /r43 | 0.0026 | 0.0009 | 2.86 | 0.0040 | 0.0008 | 0.0044 |
| /r53 | -0.0387 | 0.0052 | -7.47 | 0.0000 | -0.0489 | -0.0286 |
| /r63 | 0.0119 | 0.0067 | 1.77 | 0.0770 | -0.0013 | 0.0250 |
| /r14 | 0.0011 | 0.0080 | 0.14 | 0.8900 | -0.0146 | 0.0168 |
| /r24 | -0.0026 | 0.0062 | -0.42 | 0.6720 | -0.0147 | 0.0095 |
| /r34 | -0.0658 | 0.0092 | -7.15 | 0.0000 | -0.0838 | -0.0478 |
| /r44 | 0.0008 | 0.0012 | 0.71 | 0.4760 | -0.0015 | 0.0032 |
| /r54 | -0.0747 | 0.0062 | -11.97 | 0.0000 | -0.0869 | -0.0625 |
| /r64 | 0.0017 | 0.0074 | 0.23 | 0.8150 | -0.0128 | 0.0163 |
| **Delta** |  |  |  |  |  |  |
| /e1 | 0.3848 | 0.0150 | 25.67 | 0.0000 | 0.3554 | 0.4142 |
| /e2 | 0.3505 | 0.0185 | 18.95 | 0.0000 | 0.3143 | 0.3868 |
| /e3 | 0.1942 | 0.0140 | 13.88 | 0.0000 | 0.1668 | 0.2216 |
| /e4 | 0.2588 | 0.0129 | 20.06 | 0.0000 | 0.2335 | 0.2841 |
| Source: National Urban and Rural Household Income and Expenditures Survey (ENIGHUR) 2011-2012 | | | | | | |
