# Supplementary material for: The effects of taxing sugar-sweetened beverages in Ecuador: An analysis across different income and consumption groups
Source: PLoS One. 2020 Oct 13;15(10):e0240546. doi: 10.1371/journal.pone.0240546 (PMC7553359; doi:10.1371/journal.pone.0240546)
Supplement: S2 Table — Uncorrected model. (DOCX) [file pone.0240546.s002.docx]

**S2 Table. Uncompensated price elasticities: whole sample. Uncorrected model**

|  | Change in price |  |  |  |  |
| --- | --- | --- | --- | --- | --- |
| Change in quantity | **Milk** | **SSBs soft drinks** | **Water** | **SSBs other** | **Coffee and tea** |
| **Milk** | **-0,9863 ***** | 0,0612 *** | -0,0375 *** | -0,0851 *** | -0,0319 *** |
|  | (0,0217) | (0,0143) | (0,0092) | (0,0129) | (0,0088) |
| **SSBs soft drinks** | 0,0952 *** | **-1,2931 ***** | 0,0495 ** | -0,0111 | 0,0789 *** |
|  | (0,0234) | (0,0237) | (0,0162) | (0,0096) | (0,0119) |
| **Water** | -0,0450 ** | 0,0644 *** | **-0,9277 ***** | -0,0964 *** | -0,0272 * |
|  | (0,0155) | (0,0169) | (0,0198) | (0,0157) | (0,0106) |
| **SSBs other** | -0,1916 *** | 0,0015 | -0,1520 *** | **-0,7087 ***** | 0,0047 |
|  | (0,0444) | (0,0224) | (0,0282) | (0,0336) | (0,0181) |
| **Coffee and tea** | 0,0856 | 0,3370 *** | 0,0684 * | 0,1466 *** | **-1,0200 ***** |
|  | (0,0440) | (0,0334) | (0,0296) | (0,0293) | (0,0246) |

Source: National Survey of Income and Expenditure for Urban and Rural Households 2011- 2012. Ecuador. Bold denote own-price elasticities. Std. Err. In parentheses. * p<0.05; ** p<0.01; *** p<0.001
