# Supplementary material for: The effects of taxing sugar-sweetened beverages in Ecuador: An analysis across different income and consumption groups
Source: PLoS One. 2020 Oct 13;15(10):e0240546. doi: 10.1371/journal.pone.0240546 (PMC7553359; doi:10.1371/journal.pone.0240546)
Supplement: S3 Table — (DOCX) [file pone.0240546.s003.docx]

**S3 Table. Uncompensated price elasticities: first income quintile**

|  | Change in price | |  |  |  |
| --- | --- | --- | --- | --- | --- |
| Change in quantity | **Milk** | **SSBs soft drinks** | **Water** | **SSBs other** | **Coffee and tea** |
| **Milk** | **-1.2141 ***** | -0.1509 *** | 0.0419 | 0.3019 *** | -0.0821 *** |
|  | (0.0311) | (0.0258) | (0.0232) | (0.0246) | (0.0123) |
| **SSBs soft drinks** | -0.0858 ** | **-1.3248 ***** | 0.2456 *** | 0.0190 | 0.1437 *** |
|  | (0.0304) | (0.0431) | (0.0283) | (0.0326) | (0.0112) |
| **Water** | 0.1269 *** | 0.4007 *** | **-0.7518 ***** | -0.5018 *** | -0.1753 *** |
|  | (0.0329) | (0.0362) | (0.0395) | (0.0318) | (0.0145) |
| **SSBs other** | 0.5924 *** | -0.0288 | -0.6811 *** | **-1.1047 ***** | 0.0707 ** |
|  | (0.0431) | (0.0542) | (0.0379) | (0.0630) | (0.0217) |
| **Coffee and tea** | 0.0648 * | 0.4290 *** | -0.4256 *** | 0.0443 | **-0.9287 ***** |
|  | (0.0304) | (0.0376) | (0.0267) | (0.0383) | (0.0198) |

Source: National Survey of Income and Expenditure for Urban and Rural Households 2011- 2012. Ecuador. Bold denote own-price elasticities. Std. Err. In parentheses. * p<0.05; ** p<0.01; *** p<0.001
