# Supplementary material for: The effects of taxing sugar-sweetened beverages in Ecuador: An analysis across different income and consumption groups
Source: PLoS One. 2020 Oct 13;15(10):e0240546. doi: 10.1371/journal.pone.0240546 (PMC7553359; doi:10.1371/journal.pone.0240546)
Supplement: S4 Table — (DOCX) [file pone.0240546.s004.docx]

**S4 Table. Uncompensated price elasticities: fifth income quintile**

|  | Change in price | |  |  |  |
| --- | --- | --- | --- | --- | --- |
| Change in quantity | **Milk** | **SSBs soft drinks** | **Water** | **SSBs other** | **Coffee and tea** |
| **Milk** | **-1,1735 ***** | -0,0928 *** | 0,0458 * | 0,3333 *** | -0,0902 *** |
|  | (0,0323) | (0,0266) | (0,0228) | (0,0247) | (0,0107) |
| **SSBs soft drinks** | -0,1468 ** | **-1,3854 ***** | 0,3607 *** | 0,0567 | 0,1451 *** |
|  | (0,0453) | (0,0653) | (0,0428) | (0,0497) | (0,0170) |
| **Water** | 0,0962 ** | 0,3679 *** | **-0,7478 ***** | -0,5181 *** | -0,1636 *** |
|  | (0,0329) | (0,0371) | (0,0398) | (0,0318) | (0,0139) |
| **SSBs other** | 0,4565 *** | -0,1019 * | -0,5970 *** | **-1,1245 ***** | 0,0514 ** |
|  | (0,0377) | (0,0456) | (0,0320) | (0,0519) | (0,0166) |
| **Coffee and tea** | -0,3132 *** | 0,5393 *** | -0,6530 *** | 0,3754 *** | **-0,3224 ***** |
|  | (0,0763) | (0,0845) | (0,0699) | (0,0822) | (0,0595) |

Source: National Survey of Income and Expenditure for Urban and Rural Households 2011- 2012. Ecuador. Bold denote own-price elasticities. Std. Err. In parentheses. * p<0.05; ** p<0.01; *** p<0.001
