# Supplementary material for: The effects of taxing sugar-sweetened beverages in Ecuador: An analysis across different income and consumption groups
Source: PLoS One. 2020 Oct 13;15(10):e0240546. doi: 10.1371/journal.pone.0240546 (PMC7553359; doi:10.1371/journal.pone.0240546)
Supplement: S5 Table — (DOCX) [file pone.0240546.s005.docx]

**S5 Table. Uncompensated price elasticities: light soft drink consumers**

|  | **Change in price** | | | | |
| --- | --- | --- | --- | --- | --- |
| Change in quantity | **Milk** | **SSBs soft drinks** | **Water** | **SSBs other** | **Coffee and tea** |
| **Milk** | **-1,2052 ***** | -0,1366 *** | 0,0433 | 0,3072 *** | -0,0844 *** |
|  | (0,0312) | (0,0258) | (0,0230) | (0,0245) | (0,0117) |
| **SSBs soft drinks** | -0,1408 ** | **-1,3823 ***** | 0,3561 *** | 0,0556 | 0,1470 *** |
|  | (0,0452) | (0,0643) | (0,0422) | (0,0488) | (0,0168) |
| **Water** | 0,1172 *** | 0,3833 *** | **-0,7571 ***** | -0,4919 *** | -0,1699 *** |
|  | (0,0319) | (0,0353) | (0,0385) | (0,0309) | (0,0138) |
| **SSBs other** | 0,5196 *** | -0,0473 | -0,6151 *** | **-1,1034 ***** | 0,0557 ** |
|  | (0,0379) | (0,0474) | (0,0331) | (0,0548) | (0,0185) |
| **Coffee and tea** | -0,0184 | 0,3651 *** | -0,4869 *** | 0,1028 * | **-0,7206 ***** |
|  | (0,0397) | (0,0461) | (0,0352) | (0,0484) | (0,0285) |

Source: National Survey of Income and Expenditure for Urban and Rural Households 2011- 2012. Ecuador. Bold denote own-price elasticities. Std. Err. In parentheses. * p<0.05; ** p<0.01; *** p<0.001.
