# Supplementary material for: The effects of taxing sugar-sweetened beverages in Ecuador: An analysis across different income and consumption groups
Source: PLoS One. 2020 Oct 13;15(10):e0240546. doi: 10.1371/journal.pone.0240546 (PMC7553359; doi:10.1371/journal.pone.0240546)
Supplement: S6 Table — (DOCX) [file pone.0240546.s006.docx]

**S6 Table. Uncompensated price elasticities: heavy soft drinks consumers**

|  | Change in price | | | | |
| --- | --- | --- | --- | --- | --- |
| Change in quantity | **Milk** | **SSBs soft drinks** | **Water** | **SSBs other** | **Coffee and tea** |
| **Milk** | **-1,2908 ***** | -0,0080 | 0,1306 ** | 0,6256 *** | -0,2568 *** |
|  | (0,0595) | (0,0494) | (0,0412) | (0,0448) | (0,0188) |
| **SSBs soft drinks** | -0,0325 * | **-1,2746 ***** | 0,1343 *** | -0,0207 | 0,1432 *** |
|  | (0,0149) | (0,0215) | (0,0141) | (0,0165) | (0,0056) |
| **Water** | 0,1261 * | 0,5408 *** | **-0,5994 ***** | -0,8394 *** | -0,1740 *** |
|  | (0,0537) | (0,0616) | (0,0645) | (0,0519) | (0,0234) |
| **SSBs other** | 0,5402 *** | -0,1861 ** | -0,7632 *** | **-1,1895 ***** | 0,0894 *** |
|  | (0,0538) | (0,0637) | (0,0449) | (0,0707) | (0,0228) |
| **Coffee and tea** | -0,2194 | 3,0411 *** | -2,0167 *** | 1,0461 *** | **-1,6036 ***** |
|  | (0,1200) | (0,1450) | (0,1097) | (0,1419) | (0,0940) |

Source: National Survey of Income and Expenditure for Urban and Rural Households 2011- 2012. Ecuador. Bold denote own-price elasticities. Std. Err. In parentheses. * p<0.05; ** p<0.01; *** p<0.001.
